# T329S Mutation in the *FMO3* Gene Alleviates Lipid Metabolic Diseases in Chickens in the Late Laying Period

**DOI:** 10.3390/ani12010048

**Published:** 2021-12-27

**Authors:** Jianlou Song, Mingyi Huang, Xuefeng Shi, Xianyu Li, Xia Chen, Zhaoxiang He, Junying Li, Guiyun Xu, Jiangxia Zheng

**Affiliations:** 1 College of Animal Science and Technology, China Agricultural University, Beijing 100193, China; songjianlou@163.com (J.S.); myhuang96@126.com (M.H.); xueqian_happy@163.com (X.S.); xianyuli96@163.com (X.L.); ahknow@163.com (Z.H.); lijunying@cau.edu.cn (J.L.); ncppt@cau.edu.cn (G.X.); 2 Institute of Animal Husbandry and Veterinary Medicine, Beijing Academy of Agriculture and Forestry Sciences, Beijing 100094, China; chenxia_91@163.com

**Keywords:** flavin-containing monooxygenase 3, mutation, lipid metabolic disease, chicken, health status

## Abstract

**Simple Summary:**

The lipid deposition and health status of egg-laying hens is crucial to the development of the poultry industry. This study aimed to evaluate the effects of genetic variations in the flavin-containing monooxygenase 3 (*FMO3*) on the lipid metabolic diseases of laying hens during the late laying period. The results showed that the T329S mutation in *FMO3* moderated the lipid parameters and decreased the atherosclerotic lesions and hepatic steatosis in laying hens with homozygous T329S mutation. In conclusion, the T329S mutation in *FMO3* is closely associated with the improvement of lipid metabolic diseases in laying hens during the late laying period. The results of this study may contribute to overcoming the challenge of lipid metabolic diseases in laying hens during the late laying period.

**Abstract:**

The T329S mutation in flavin-containing monooxygenase 3 (*FMO3*) impairs the trimethylamine (TMA) metabolism in laying hens. The TMA metabolic pathway is closely linked to lipid metabolic diseases, such as atherosclerosis and fatty liver disease. We aimed to evaluate the effects of the T329S mutation in *FMO3* on lipid metabolism in chickens during the late laying period. We selected 18 *FMO3* genotyped individuals (consisting of six AA, six AT, and six TT hens) with similar body weight and production performance. The lipid metabolism and deposition characteristics of the laying hens with different genotypes were compared. The T329S mutation moderated the serum-lipid parameters in TT hens compared to those in AA and AT hens from 49 to 62 weeks. Furthermore, it reduced the serum trimethylamine N-oxide concentrations and increased the serum total bile acid (*p* < 0.05) and related lipid transporter levels in TT hens. Moreover, it significantly (*p* < 0.01) decreased atherosclerotic lesions and hepatic steatosis in TT hens compared to those in the AA and AT hens. Our findings may help improve the health status in laying hens during the late laying period.

## 1. Introduction

Flavin-containing monooxygenases (FMOs, EC 1.14.13.8) are an important class of oxidases that are responsible for the oxygenation of soft nucleophilic heteroatom-containing organic substances, such as nitrogen, sulfur, and phosphorous. Accordingly, FMOs play a significant role in the metabolism and detoxification of drugs, endogenous substances, and dietary-derived compounds [1]. *FMO3*, which plays an important role in the metabolism of endogenous substances, is the most important member of the FMO family and is the predominant isoform that is involved in the trimethylamine (TMA) metabolic pathway [2]. This pathway involves the oxidation of TMA to form trimethylamine N-oxide (TMAO) by hepatic *FMO3*, which is closely linked to many metabolic characteristics [3]. It has been reported that some mutations in the *FMO3* gene in humans can either abolish or diminish the catalytic activity of the enzyme and inhibit the oxidization of TMA, a fishy odor substance, which results in the fish-odor syndrome [4]. Similarly, a threonine to serine substitution at the 329th position (T329S, *FMO3* c.984 A > T) of *FMO3* was detected in chickens, whose function is similar to those of humans and associated with the fishy-odor eggs traits [5,6]. Thus, we speculated that the T329S could decrease the circulating TMAO levels in homozygous T329S (TT) hens compared to those of homozygous wild-type (AA) hens. This mutation has a low mutation frequency in several Chinese local chicken varieties, such as Huainan Mahuang hens (3.8%), Hebei Chai hens (6.8%), and Wenchang hens (9.6%) [7,8]. In parallel, it was also detected in CAU-3, a strain of brown-egg dwarf chickens, that reaches to 5% production at 21 weeks of age and lays approximately 180 eggs from hatch to 52 weeks.

Recently, a novel function of the TMA metabolic pathway on lipid metabolic diseases in mammals was identified [9,10,11,12]. TMAO was first suggested as an independent marker for atherosclerosis (AS), and dietary TMAO supplementation confirmed its role in promoting the development of AS and thrombosis in mice [9,10]. Furthermore, the circulating TMAO levels have been associated with adverse effects on fatty liver disease (FLD), and increased liver inflammation and damage have also been reported in human studies [11,12]. A possible mechanism underlying the involvement of TMAO in AS and FLD pathogenesis has been proposed, where TMAO affects lipid absorption and cholesterol homeostasis by decreasing the total bile acid (TBA) pool size [13,14]. Hepatic *FMO3* plays a direct role in the cholesterol and triglycerides (TGs) metabolism in hyperlipidemic mice [15]. Further, Hepatic *FMO3* has been demonstrated to be a central regulator of hepatic cholesterol balance by regulating reverse cholesterol transport (RCT), in mouse models [16]. In mice, the natural variability of *FMO3* not only reduces TMAO production but also stimulates the RCT process to promote cholesterol efflux [15]. There is increasing evidence that supports the role TMA metabolic pathway in hyperlipidemia, AS, and FLD, and that the *FMO3* genetic variants may have a positive effect on improving lipid metabolism in mammals [15,16,17].

It has been suggested that the regulation of cholesterol disposal in poultry is highly similar to that in mammals [18]; however, the effect of the TMA metabolic pathway on lipid metabolic disease in poultry remains unclear. Lipid metabolic diseases, such as AS and FLD, negatively impact the poultry industry, as they reduce the production performance of laying hens in the late laying period [19,20]. They can even induce sudden death, resulting in major economic losses [21]. Previous studies have demonstrated that FLD can cause up to 5% mortality in commercial layers during the laying cycle and 74% of the total mortality in caged laying hens in Queensland, Australia. Meanwhile, the mortality of laying hens with AS has not been reported [22,23]. The occurrence and development of these diseases are associated with adiposity in the laying process [19,20]. To meet the needs of egg yolk formation during the egg-laying period, hepatic lipid synthesis and metabolism are strongly activated, resulting in elevated levels of TG and phospholipids. Under the action of multiple lipid transfer proteins, these lipids are assembled into very low density lipoprotein (VLDL) particles that are transported to the follicle [18,24]. For this reason, and coupled with the extension of the laying cycle from the first 72–80 weeks, some breeding companies have even prolonged the laying age to 100 weeks [25,26]. Continuous egg production with a high dietary consumption of carbohydrate can markedly induce adiposity, increasing the risk of FLD, as well AS [25,27]. Several studies have demonstrated that an increasing level of bile acid production or an exogenous bile acid intake can play a dramatic role in promoting lipid absorption and cholesterol excretion for improving this situation [28,29,30,31]. However, until now, the impacts of SNPs in *FMO3* have been largely unstudied regarding lipid metabolism in chickens, other than fishy taint in eggs.

Given the alleviating effects of *FMO3* mutations in the TMA metabolic pathway on lipid metabolic diseases, we hypothesized that the TMA-metabolic-pathway-involved *FMO3* genetic variants may have a positive effect on improving the health status in chickens. In chickens, only the T329S mutation in *FMO3* is highly associated with the TMA metabolic pathway [5]. In this study, we compared the lipid metabolism and deposition characteristics among laying hens with different genotypes in the late laying period. We aimed to investigate whether the T329S mutation in *FMO3* can improve the health status by modulating the lipid metabolism in laying hens.

## 2. Materials and Methods

### 2.1. Birds and Diet

A pure line of CAU-3, maintained mainly for egg production for over 10 years in the Poultry Genetic Resource and Breeding Experimental Unit of China Agricultural University [32,33], was used in this study. This population was produced from pedigree mating, using 50 sires and 7 to 8 dams per sire. After the strain of CAU-3 was established, each generation was “bred” by a random process.

At 47 weeks of age, a total of 208 CAU-3 healthy hens were selected for blood sampling via wing vein puncture. Blood samples were collected for genotype analysis (A/T polymorphism at position 1034 of the chicken *FMO3* exon 7, chromosome 8, accession number: AJ431390). Each hen was fitted with a leg ring marked with a unique identification number. A polymerase chain reaction-restriction fragment length polymorphism assay, as described in Reference [7], was used to determine the individual *FMO3* genotypes (AA, AT, and TT) at this position. During the two-week acclimation period, 18 *FMO3* genotyped individuals (consisting of six AA, six AT, and six TT hens) with similar body weight and egg production were selected and raised in individual cages (cage size: 45 cm × 45 cm × 45 cm). The formal experimental period ranged from 49 to 62 weeks of age. During the experimental period, hens with these genotypes were fed a basal diet, which was formulated to meet the National Research Council requirements (NRC, 1994) [34], and were offered in mash form ad libitum, with each hen ingesting approximately 100 g/day. The composition and nutrient levels of the basal diet are shown in Table S1. Water was supplied by nipple drinkers. Egg production was recorded daily for each hen. The room temperature was maintained between 22 and 26 °C, and light exposure was controlled with a light/dark cycle of 16:8 h. Illumination was provided by incandescent lamps with an intensity of 10 lx (at bird-head level). All procedures, as well as the care, housing, and handling of the animals, were conducted according to accepted commercial management practices. All birds remained healthy during the feeding period. No birds were culled, and none received any medical intervention. The animal experiment was conducted in the Experimental Unit for Poultry Genetic Resource and Breeding of China Agricultural University.

### 2.2. Sample Collection

At 49 weeks of age, after an 8-h fast, blood samples were collected from all 18 genotyped individuals in the morning. At 62 weeks of age, after an 8 h fast, blood samples were collected from the same individuals in the morning. Blood samples were stored in a vacuum blood-collection tube without anticoagulant. The serum was separated by centrifugation at 3000× *g* for 15 min and stored at −20 °C until analysis. Then, the six AA, six AT, and six TT hens were humanely euthanized. The whole aorta and liver tissues were isolated and fixed in formalin for 48–72 h, before being processed for AS lesion analysis and liver histopathological observation.

In addition, three healthy AA pullets (18 weeks old) were sacrificed, and the whole aorta and liver tissues were isolated in the same manner. These samples were used as a negative control for AS lesion analysis and liver histopathological observation, since our pre-experiment found that lipid droplets were scarcely observed in the aortic wall and liver of pullets at 18 weeks.

### 2.3. Serum Index Measurement

#### 2.3.1. Serum-Lipid Parameters and Bile Acid

Serum TG, total cholesterol (TC), low-density lipoprotein cholesterol (LDL-C), high-density lipoprotein cholesterol (HDL-C), and TBA levels were determined by using commercial kits (Shanghai Jingkang Bioengineering Co., Ltd., Shanghai, China). The automatic biochemical analyzer used was a KHB ZY-1280 manufactured by the Shanghai Kehua Bio-engineering Corporation (Shanghai, China).

#### 2.3.2. Nontraditional Serum-Lipid Parameters

Nontraditional serum-lipid parameters were calculated as previously described in Reference [35]: non-HDL-C = (total cholesterol minus high-density lipoprotein cholesterol); TC/HDL-C = (total cholesterol/high-density lipoprotein cholesterol); atherogenic index (AIS) = [(total cholesterol minus high-density lipoprotein cholesterol)/high-density lipoprotein cholesterol]; lipoprotein combined index (LCI) = (total cholesterol × total TG × low-density lipoprotein cholesterol/high-density lipoprotein cholesterol).

#### 2.3.3. Serum TMA and TMAO

Pretreatment serum was prepared as previously described in Reference [36], with minor modifications. In brief, 500 μL of the serum was immediately acidified upon collection with 500 μL of hydrochloric acid (0.01 M), vortex mixed for 30 s, and stored at −20 °C until analysis of TMA and TMAO. The serum TMA and TMAO levels were measured by using headspace gas chromatography–mass spectrometry (GC–MS), as previously described in Reference [6]. A Shimadzu GC-2010 Plus gas chromatograph (Shimadzu, Kyoto, Japan) fitted with a 60 m × 0.20 mm ID fused silica capillary column coated with a 1.12 μm film of HP-VOC (Agilent Technologies, CA, USA) and a Shimadzu TQ8040 mass spectrometer were used. The detection was performed on a Shimadzu GCMS-QP2010 Plus system (Shimadzu Technologies, Kyoto, Japan) equipped with an HS-20 headspace sampler.

#### 2.3.4. Lipid-Related Transporters and VLDL

Serum protein concentrations of phospholipid transfer protein (PLTP), lecithin–cholesterol acyltransferase (LCAT), cholesterol ester transfer protein (CETP), and serum VLDL concentrations were measured by using chicken PLTP, LCAT, CETP, and VLDL enzyme-linked immunosorbent assay kits from Shanghai Jingkang Bioengineering Co., Ltd. (PLTP ELISA kit JLC23302, LCAT ELISA kit JLC10740, CETP ELISA kit JLC23020, and VLDL ELISA kit JLC10779), following the manufacturer’s instructions. The antigen, which the VLDL ELISA kit measured, is apolipoprotein B (ApoB), a fraction of VLDL particles. VLDL levels were calculated from a standard curve, which was plotted according to the OD of the concentration of standards (Standard concentration was followed by 16, 8, 4, 2, 1, and 0.5 mmol/L).

### 2.4. Lipid-Deposition Characteristics

#### 2.4.1. AS Lesions Analysis

AS lesions were quantified by using en face analysis of the aorta (including the aortic arch, thoracic, and abdominal regions) and cross-sectional analysis of the aortic arch, as previously described in References [37,38], with minor modifications. For en face analysis, the aorta was longitudinally opened and stained with Oil red O (Wuhan Service Biotechnology Co., Ltd., Wuhan, China) to detect lipids and to determine the lesion area. AS lesions of the aorta were expressed as percentages of the total surface area. For cross-sectional analysis, a small segment of the aortic arch (in the same area) was embedded in OCT compound (Sigma-Aldrich, St. Louis, MO, USA) and frozen at −20 °C. Sections (8 µm thick) were collected. Lesions from ten alternating sections were stained with Oil Red O and hematoxylin. For each section of the aortic arch, ten randomly selected areas were assessed, using light microscopy at ×40 magnification.

#### 2.4.2. Liver Histopathological Observation

Liver sections were examined for steatosis, using Oil Red O staining, as previously described in Reference [39], with minor modifications. For cryo-section cutting, fixed samples were embedded in frozen OCT (Sigma-Aldrich, St. Louis, MO, USA) and then sectioned at 10 µm; all operations were carried out under frozen conditions. Samples were then stained with Oil Red O (Wuhan Service Biotechnology Co., Ltd., Wuhan, China), differentiated with isopropanol, washed with distilled water, and stained with hematoxylin. For each section of the liver, 10 randomly selected areas were assessed by using light microscopy at ×80 magnification.

Images of aortic en face, aortic arch cross-sections, and liver sections were taken by using a Canon EOS 7D digital camera (Canon, Tokyo, Japan), and the aorta, aortic arch, and liver lesions were quantified by using computer-assisted image analysis (ImageJ, NIH Image, National Institutes of Health, version 1.8.0), according to procedures described in Reference [40].

### 2.5. Statistical Analysis

The genotype distributions in the flock were tested for Hardy–Weinberg equilibrium. Statistical analyses were conducted by using the R software (version 4.0.3), and the figures were plotted by using GraphPad Prism (version 7.04; GraphPad Software, San Diego, CA, USA). Due to the small sample size, nonparametric procedures were used. Basic descriptive statistics: An independent-sample Kruskal–Wallis test was used to analyze the average number of laid eggs, the serum indices, and the lipid-deposition characteristics, including aortic lesion (AL), aortic arch lesion (AAL), and hepatic lipid deposition (HLD), in chickens with different genotypes. Rate of change (RC) of serum indices of chickens in the same genotype between 49 and 62 weeks of age was obtained by applying the following formula:

RC (%) = (X_62_ − X_49_)/X_49_ × 100%,

where X_62_ includes the levels of the serum TG, TC, LDL-C, HDL-C, and TBA of chickens at 62 weeks; and X_49_ includes the levels of the serum TG, TC, LDL-C, HDL-C, and TBA of chickens at 49 weeks, corresponding to those of 62 weeks.

In addition, correlations between nontraditional serum-lipid parameters (AIS, LCI, etc.) and lipid-deposition characteristics, as well as correlations between TMAO, TMA, TBA, and lipid-deposition characteristics, were analyzed by using Pearson’s correlation coefficient. The *p*-Values < 0.05 and < 0.01 were considered statistically significant and extremely statistically significant, respectively. Data are expressed as the mean ± standard deviation.

## 3. Results

### 3.1. Hardy–Weinberg Equilibrium Test and Egg Production

A total of 151 AA, 51 AT, and six TT hens were obtained from the whole flock. The genotype frequencies of *FMO3* were 72.59% (AA), 24.52% (AT), and 2.88% (TT), which complied with the Hardy–Weinberg equilibrium (*p* = 0.59). The egg production of the 18 sampling chickens from 49 to 62 weeks showed that TT hens produced 52 eggs each, which was higher than (*p* > 0.05) the number of eggs produced by AA (49.8 eggs per hen) and AT (47.2 eggs per hen) hens.

### 3.2. Serum-Lipid Parameter and TBA Levels

The changes in serum-lipid parameters and TBA levels in laying hens at the ages of 49 and 62 weeks are shown in Table 1. The serum-lipid parameter levels, including TG, TC, LDL-C, and HDL-C, and the serum TBA levels were all increased at 62 weeks, compared to those at 49 weeks, regardless of the genotype of the chickens. However, the T329S mutation in *FMO3* prominently (*p* < 0.05) decreased the RC of TG, TC, and LDL-C levels in TT hens. In addition, at 62 weeks, as an effect of the T329S mutation, the serum TG and LDL-C levels were decreased, especially the serum TG levels (*p* < 0.05) in AT and TT hens compared to those of AA hens. Additionally, the T329S mutation of *FMO3* decreased the serum TC levels (*p* < 0.05) but increased the serum TBA levels (*p* < 0.05) in TT hens compared to those of AA and AT hens.

### 3.3. Metabolic Characteristics

The metabolic characteristics of chickens for the AA, AT, and TT genotypes at the age of 62 weeks are shown in Table 2. As a result of the T329S mutation, the nontraditional lipid parameters, including non-HDL-C, TC/HDL-C, AIS, and the LCI ratios, in TT hens were decreased compared to those of AA and AT hens. In particular, the AIS decreased by 30% and 40% (*p* < 0.05), and the LCI decreased by 70% and 35% (*p* < 0.05), respectively, compared to those of AA and AT hens. The serum TMAO concentration was also reduced by approximately 12%, with increasing serum TMA concentrations in TT hens compared to those of AA and AT hens, although the difference was not statistically significant (*p* > 0.05). Moreover, the T329S mutation in TT hens increased the serum PLTP, LCAT, CETP, and VLDL concentrations by approximately two-fold (*p* < 0.01) compared to those of AA and AT hens.

### 3.4. Lipid-Deposition Characteristics

**Figure 1:**
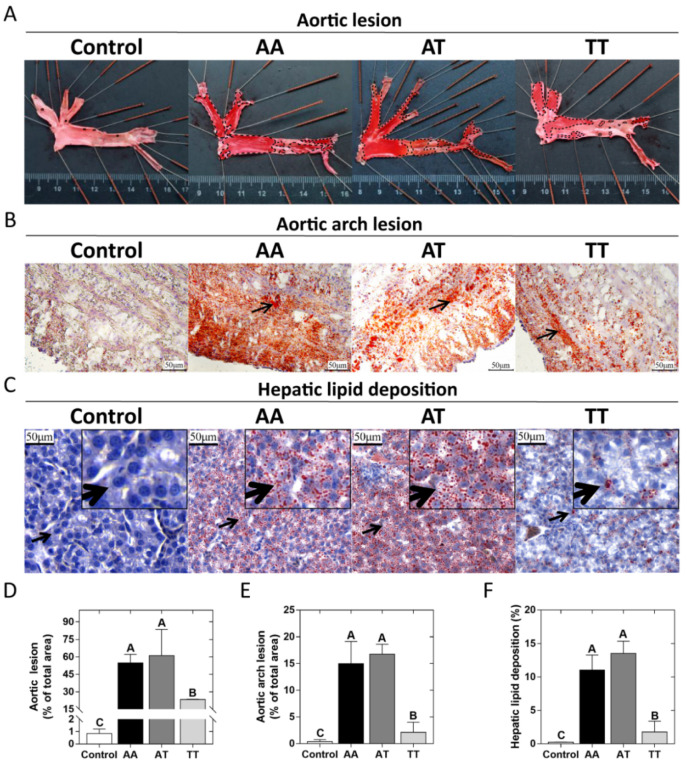
Pathological observations of lipid deposition in the aortic wall and liver in different genotyped chickens. (**A**) Oil Red O staining of the whole aorta with aortic lesions (black dashed lines). (**B**) Oil Red O and hematoxylin staining of aortic arch cross-sections, with arrows point to lipid droplets. (**C**) Oil Red O and hematoxylin staining of liver sections, with arrows point to local tissue magnification. (**D**–**F**) Quantification of staining results (% of total area) of the aorta, aortic arch cross-section, and liver (corresponding to **A**–**C**), respectively. Values are expressed as means ± SD, *n* = 6 hens each genotype. ^A,B^ Means within a histogram with no common superscripts differ significantly (*p* < 0.01).

**Figure 2:**
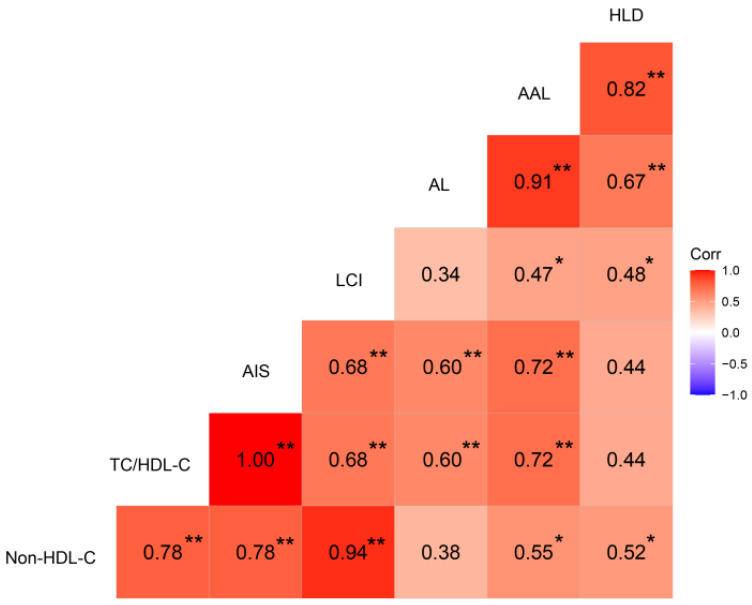
Heat map based on Pearson’s correlations for the relationship between nontraditional lipid parameters (AIS, LCI, etc.) and aortic lesion, aortic arch lesion, and hepatic lipid deposition in chickens. The color scale represents Pearson’s correlation coefficients, with red and bluish violet representing positive and negative correlations, respectively. Ranging from 1.0 (maximum positive correlation) to −1.0 (maximum anti-correlation), with 0 indicating no correlation. Non-HDL-C, non-HDL-C = TC − HDL-C; TC, total cholesterol; HDL-C, high-density lipoprotein cholesterol; AIS, AIS = (TC − HDL-C)/HDL-C; LCI, LCI = TC × TG × LDL-C/HDL-C; TG, triglyceride; LDL-C, low-density lipoprotein cholesterol; AL, aortic lesion; AAL, aortic arch lesion; HLD, hepatic lipid deposition. * *p* < 0.05, ** *p* < 0.01.

The pathological observations of lipid deposition in the aortic wall and liver of chickens for the AA, AT, and TT genotypes are shown in Figure 1. Compared to the controls (AA pullets, 18 weeks), all hens had varying degrees of lipid deposition in the aorta, aortic arch, and hepatocytes at 62 weeks (Figure 1AB). However, it was noticeable that the aorta and aortic arch cross-section lesion proportion in TT hens was less (*p* < 0.01) than half of that in AA and AT hens as an effect of the T329S mutation in *FMO3* (Figure 1A,B,D,E). Oil Red O staining of the liver showed that TT hens had the least (*p* < 0.01) hepatic lipid droplet accumulation compared to that of AA and AT hens (Figure 1C,F). In addition, the trends of AL, AAL, and HLD of the 18 chickens were all positively correlated with the nontraditional lipid parameters (AIS, LCI, etc.), as shown in Figure 2.

### 3.5. Correlations among the Serum TMAO, TMA, and Lipid-Deposition Characteristics

**Figure 3:**
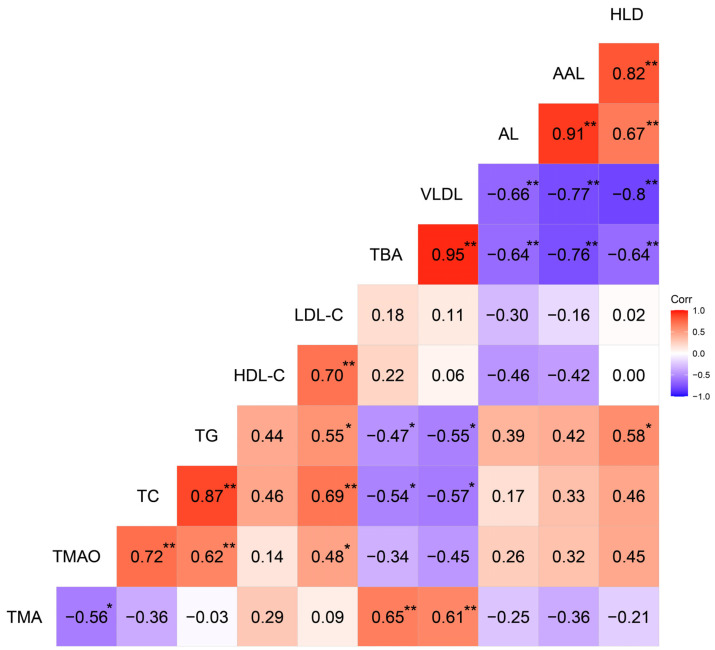
Heat map based on Pearson’s correlations for the relationship between serum trimethylamine N-oxide, trimethylamine, and lipid-deposition characteristics in the selected 18 hens at 62 weeks. The color scale represents Pearson’s correlation coefficients, with red and bluish violet representing positive and negative correlations, respectively. Ranging from 1.0 (maximum positive correlation) to −1.0 (maximum anti-correlation), with 0 indicating no correlation. TMA, trimethylamine; TMAO, trimethylamine N-oxide; TC, total cholesterol; TG, triglyceride; HDL-C, high-density lipoprotein cholesterol; LDL-C, low-density lipoprotein cholesterol; TBA, total bile acid; VLDL, very low density lipoprotein; AL, aortic lesion; AAL, aortic arch lesion; HLD, hepatic lipid deposition. * *p* < 0.05, ** *p* < 0.01.

The correlation matrix of the relationships among serum TMAO, TMA, TBA, and lipid-deposition characteristics is shown in Figure 3. Of the 18 samples, the serum TMAO levels were positively (*p* > 0.05) correlated with AL (*r* = 0.26), AAL (*r* = 0.32), and HLD (*r* = 0.45), but negatively (*p* > 0.05) correlated with the serum TBA levels (*r* = −0.34). Serum TBA levels were negatively (*p* < 0.01) correlated with AL (*r* = −0.64), AAL (*r* = −0.76), and HLD (*r* = −0.64), whereas they were positively (*p* < 0.01) correlated with serum VLDL (*r* = 0.95) levels.

## 4. Discussion

The present study showed that the T329S mutation in *FMO3* was associated with the improvement of lipid metabolic diseases in TT hens during the late laying period. This result could be attributed to the T329S mutation moderating the serum-lipid parameters, reducing TMAO production, and alleviating lipid deposition in TT hens. The present study proposed a new role of the T329S mutation in improving the health status in egg-laying hens. Previous studies have focused on the fact that feeding TT hens a high-level TMA precursor diet increases the risk of fish-odor eggs [5,6]. The effect of the T329S mutation on lipid metabolism and health status has not yet been considered. To the best of our knowledge, our study is the first to investigate the effect of the T329S mutation in *FMO3* on health status in laying hens. Our results may contribute to overcoming the challenges of lipid metabolic diseases in laying hens during the late laying period.

In chickens, the lipid metabolic diseases, such as AS and FLD, primarily occur in the late laying period and are mainly caused by the imbalance between deposition and removal of lipids [21,41]. Serum-lipid parameters, such as TG, TC, and LDL-C, are the direct indicators of lipid metabolism balance during the laying period [42]. In this study, the increases in serum TG and TC levels in TT hens were less than those in AA and AT hens, and the increases in serum LDL-C in TT hens were less than those in AA hens from 49–62 weeks. TG, TC, and LDL-C are all major risk factors for AS and FLD [43,44]. Persistently high levels of serum TG, TC, and LDL-C can increase the risk of AS and FLD, resulting in the decreased production performance of laying hens and even death [45]. In contrast, modulating the TG and cholesterol levels can reduce the incidence of these diseases in aged laying hens [21]. Thus, more stable lipid parameters could be one of the origins of health status improvement in TT hens. In parallel, the stability of lipid parameters could be attributed to the action of the T329S mutation. In our study, the decreases of the TG and LDL-C levels by T329S in both TT and AT (*FMO3*, c. 984 A > T) hens at 62 weeks confirmed this assumption. This is similar to the findings of Reference [41], who showed that T329S upregulated the mRNA levels of genes involved in cholesterol and TG transport (e.g., apovitellenin 1, and ATP binding cassette transporters G5 and G8) in TT hens. Therefore, we assumed that the T329S mutation in *FMO3* could moderate the changes in lipid parameter levels.

Compared with the conventional lipid parameters, the non-HDL-C, TC/HDL-C, AIS, and LCI ratios are better indicators of AS, a disease that is positively associated with FLD [46]. These ratios have been suggested to be accurate predictors of AS [47]. For example, a lower AIS ratio in healthy controls (3.19) than that in the disease onset group (3.51) indicated a low risk [35]. In our study, the decreases of non-HDL-C, TC/HDL-C, AIS, and LCI ratios demonstrated that the T329S mutation in *FMO3* had an anti-AS effect on TT hens to some extent, as these ratios were positively correlated with AL, AAL, and HLD in chickens (Figure 2). The least aortic and aortic arch lesions in TT hens (Figure 1A,B; *p* < 0.01) provided evidence for this conclusion. At the same time, our results also showed that TT hens had only mild degrees of hepatic steatosis (Figure 1C; *p* < 0.01), thus indicating a lower risk of FLD in TT hens. Therefore, these results demonstrated that the T329S mutation could reduce the risk of AS lesions and FLD in laying hens.

**Figure 4:**
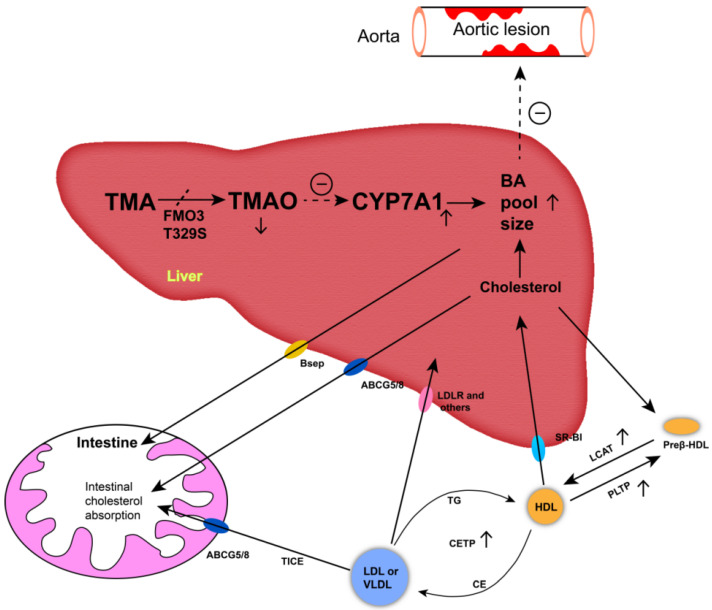
Schematic representation of pathways of the T329S mutation in flavin-containing monooxygenase 3 (FMO3) associated with the metabolic characteristics in chickens. Black arrows indicate movement of TMA/TMAO and cholesterol through the body. Blunt-end arrows indicate activation (+) or inhibition (−) of specified receptors and transporters or pathways. An increased bile acid pool size can alleviate the development of aortic lesions (㊀). Mutation T329S diminishes the ability of FMO3 to oxidize trimethylamine (TMA) to trimethylamine N-oxide (TMAO) and decreases the circulating TMAO levels (↓), thus decreasing the inhibition of cholesterol 7α-hydroxylase (CYP7A1) expression (↑), accordingly. The upregulation of rate-limiting enzyme increases the levels of bile acid (BA,↑). The preferential precursor for BA biosynthesis is high-density lipoprotein cholesterol (HDL-C). HDL-C needs to be delivered to hepatocytes by high-density lipoprotein (HDL) particles. The formation of mature HDL requires the action of phospholipid transfer protein (PLTP) and lecithin: cholesterol acyltransferase (LCAT). HDL cholesterol esters can be transferred to very low density lipoprotein (VLDL) or low-density lipoprotein (LDL) by the cholesteryl ester transfer protein (CETP) and be returned to the liver through the low-density lipoprotein receptor (LDLR) or other LDL and VLDL receptors. Preβ-HDL, preβ-high-density lipoprotein particles; SR-BI, scavenger receptor class B type I; ABCG5/G8, ATP binding cassette transporters G5 and G8; Bsep, bile salt export pump; TICE, trans-intestinal cholesterol export route—promotes the flow of cholesterol from plasma to enterocytes and the intestinal lumen.

The decreased AS lesion and hepatic steatosis could be attributed to the decrease of circulating TMAO concentrations in TT hens. Mutation T329S diminishes the ability of FMO3 to oxidize TMA to TMAO [5,48]. Thus, circulating TMAO, the oxide of TMA, was reduced in TT hens. Previous studies have suggested that circulating TMAO levels are adversely associated with AS and fatty liver events, the increase of which could promote the formation of AS and FLD [10,11,12]. In our study, there was no significant (*p* > 0.05) correlation between TMAO concentrations and AL, AAL, or FLD in the chicken model, meaning that there was no direct association between TMAO concentrations and these diseases. However, the observations related to the bile acids could provide an alternative mechanism for the differences observed in AL, AAL, and FLD among different genotyped chickens. It is generally accepted that TMAO inhibits bile acid synthesis by decreasing cholesterol 7α-hydroxylase expression [13]. Bile acid synthesis and excretion are the major pathways of cholesterol and lipid catabolism (see Figure 4 for details). The obstruction of bile acid synthesis limits the efflux of cholesterol and increases lipid deposition, resulting in the occurrence and development of AS and fatty liver [13,14]. In contrast, the reduction of TMAO increases the synthesis of bile acids, and this increase can alleviate lipid deposition [38]. In our study, reduced TMAO concentrations were associated with increased serum TBA concentrations in TT hens. The negative correlation between TMAO and TBA concentrations supports the interpretation that T329S can promote bile acid synthesis due to reductions in TMAO concentrations in TT hens. Moreover, the preferential precursor for bile acid biosynthesis is HDL-C [49]. HDL-C needs to be delivered to hepatocytes by high-density lipoprotein (HDL) particles, a process mediated by scavenger receptor class B type I. The formation of mature HDL requires the action of PLTP and LCAT [13,50]. In TT hens, the T329S mutation significantly (*p* < 0.01) increased the serum PLTP and LCAT protein concentrations. However, the serum level of HDL-C in TT hens did not decrease. Therefore, we suggest that T329S increased bile acid synthesis by reducing circulating TMAO concentrations and subsequently improving RCT in laying hens.

Additionally, our study also detected that the serum CETP protein and VLDL concentrations were increased in TT hens. CETP can transfer HDL cholesterol esters (CEs) into VLDL or LDL [50]. The increased CETP may be a response to meet the demand for more CE uptake for the increased VLDL, which has already been secreted from the liver in TT hens. The major VLDL classes present in laying hens are the yolk-targeted small-diameter triacylglycerol-rich particles (VLDLy). VLDLy delivers all its triacylglycerol intact to the oocyte, which finally develops into the mature egg yolk [24]. The elevation of serum VLDL in TT hens may attribute to an increase in demand for VLDLy assembly, since it was detected that TT hens had an increasing trend in production performance in our study. A previous study reported that T329S increased the expression of apolipoprotein VLDL-II, a specific apolipoprotein of VLDLy [41], may support this statement. The association between T329S mutation and the process of yolk formation should be further explored. At the same time, the elevation of hepatic VLDL secretion may protect the liver from TG accumulation [41,51]. Consistent with it, a negative correlation (*r* = −0.55, *p* < 0.05) between serum VLDL and TG concentrations was detected in our study. However, this negative correlation does not mean that peripheral lipolysis of VLDL is increased [24]. The lack of correlation (*r* = 0.11, *p* > 0.05) between serum VLDL (ApoB) and LDL-C concentrations could just as reasonably be hypothesized to indicate that peripheral lipolysis is reduced. At the same time, it could be implied that most of the VLDL (ApoB) is being deposited in eggs not metabolized by the hen. The TT hens drive the negative correlation between VLDL and TG, and the reduction of TG may rely on the improved egg production observed in those hens. These hypotheses warrant further research.

In addition, the T329S mutation diminishes the ability of FMO3 to oxidize TMA to TMAO, and this may increase the risk of fishy eggs in TT hens when they are fed a high-level TMA precursor diet [5]. Hence, we should weigh the pros and cons and apply T329S to the poultry industry appropriately or take advantage of T329S by taking a reasonable diet in the late laying period.

## 5. Conclusions

In conclusion, we proposed an association between the T329S mutation in *FMO3* and lipid metabolic diseases during the late laying period. Our analysis of the lipid metabolism and deposition characteristics showed that the T329S mutation could alleviate AL, AAL, and FLD in laying hens, and this could, in part, through downregulation, reduce the circulating TMAO level concentrations in association with increased circulating bile acids, revealing new insights into the role of T329S in association with health-state improvements in laying hens.

## Figures and Tables

**Table 1:** Serum physiological parameters of laying hens with different *FMO3* genotypes at 49 and 62 weeks of age.

Item ^1^		AA	AT	TT	Total *p*-Value
TG (mmol/L)	49 weeks	7.67 ± 0.99 ^a^	6.56 ± 1.67 ^b^	8.52 ± 0.82 ^a^	*p* < 0.05
	62 weeks	23.51 ± 1.28 ^a^	20.55 ± 2.02 ^b^	17.96 ± 3.69 ^b^	*p* < 0.05
	RC (%)^2^	206.52 ± 37.35 ^a^	213.26 ± 80.17 ^a^	110.80 ± 51.74 ^b^	*p* < 0.05
TC (mmol/L)	49 weeks	3.25 ± 0.73	3.04 ± 0.51	3.46 ± 0.48	*p* > 0.05
	62 weeks	4.93 ± 0.48 ^a^	4.23 ± 0.64 ^a^	3.94 ± 0.45 ^b^	*p* < 0.05
	RC (%)	51.69 ± 26.42 ^a^	39.14 ± 24.43 ^a^	13.87 ± 23.11 ^b^	*p* < 0.05
LDL-C (mmol/L)	49 weeks	1.84 ± 0.36	1.82 ± 0.22	1.89 ± 0.29	*p* > 0.05
	62 weeks	2.53 ± 0.29 ^a^	2.14 ± 0.38 ^b^	2.33 ± 0.22 ^a,b^	*p* < 0.05
	RC (%)	37.50 ± 20.68 ^a^	17.58 ± 19.21 ^b^	23.28 ± 15.58 ^b^	*p* < 0.05
HDL-C (mmol/L)	49 weeks	0.98 ± 0.26	0.90 ± 0.15	1.04 ± 0.13	*p* > 0.05
	62 weeks	1.78 ± 0.18 ^a^	1.45 ± 0.24 ^b^	1.67 ± 0.22 ^a,b^	*p* < 0.05
	RC (%)	81.63 ± 38.92 ^a^	61.11 ± 24.23 ^b^	60.58 ± 31.32 ^b^	*p* < 0.05
TBA (μmol/L)	49 weeks	2.52 ± 0.15	2.44 ± 0.02	2.38 ± 0.12	*p* > 0.05
	62 weeks	6.35 ± 0.64 ^b^	6.35 ± 0.36 ^b^	8.53 ± 1.91 ^a^	*p* < 0.05
	RC (%)	151.98 ± 14.45 ^b^	160.25 ± 10.28 ^b^	258.40 ± 55.35 ^a^	*p* < 0.05

^1^ TG, triglyceride; TC, total cholesterol; LDL-C, low-density lipoprotein cholesterol; HDL-C, high-density lipoprotein cholesterol; TBA, total bile acid. ^2^ RC, rate of change; RC of serum physiological parameters of chickens in the same genotype between 49 and 62 weeks was obtained applying the following formula: RC (%) = (X_62_ − X_49_)/X_49_ × 100%. X_62_ includes the levels of the serum TG, TC, LDL-C, HDL-C, and TBA of chickens at 62 weeks; X_49_ includes the levels of the serum TG, TC, LDL-C, HDL-C, and TBA of chickens at 49 weeks, corresponding to those of 62 weeks. AA, AA-genotype hens; AT, AT-genotype hens; TT, TT-genotype hens. Values are expressed as means ± SD, *n* = 6 hens each genotype. ^a,b^ Means within a row with no common superscripts differ significantly (*p* < 0.05).

**Table 2:** Metabolic characteristics of laying hens at the age of 62 weeks.

Item ^1^	AA	AT	TT	Total *p*-Value
Nontraditional lipid parameters				
Non-HDL-C (mmol/L)	3.15 ± 0.39 ^a^	2.78 ± 0.59 ^a,b^	2.27 ± 0.45 ^b^	*p* < 0.05
TC/HDL-C	2.77 ± 0.25 ^a^	2.95 ± 0.48 ^a,b^	2.38 ± 0.35 ^b^	*p* < 0.05
AIS	1.77 ± 0.25 ^a^	1.95 ± 0.48 ^a^	1.38 ± 0.35 ^b^	*p* < 0.05
LCI	166.54 ± 34.61 ^a^	133.41 ± 55.33 ^a^	98.72 ± 21.96 ^b^	*p* < 0.05
Serum TMA and TMAO levels				
TMAO (μg/mL)	7.49 ± 1.60	7.42 ± 1.01	6.62 ± 0.69	*p* > 0.05
TMA (μg/mL)	3.21 ± 0.36	3.28 ± 0.23	3.63 ± 0.38	*p* > 0.05
Lipid-related transporters				
PLTP (ng/mL)	38.86 ± 9.38 ^B^	39.89 ± 12.96 ^B^	61.35 ± 7.24 ^A^	*p* < 0.01
LCAT (mIU/mL)	9.72 ± 1.68 ^B^	10.74 ± 2.51 ^B^	18.86 ± 5.01 ^A^	*p* < 0.01
CETP (ng/mL)	1026.77 ± 150.38 ^B^	1172.33 ± 399.03 ^B^	2086.33 ± 858.76 ^A^	*p* < 0.01
VLDL (mmol/L)	3.36 ± 0.45 ^B^	3.64 ± 0.75 ^B^	7.50 ± 2.25 ^A^	*p* < 0.01

^1^ Non-HDL-C = TC − HDL-C; TC, total cholesterol; HDL-C, high-density lipoprotein cholesterol; AIS = (TC − HDL-C)/HDL-C; LCI = TC × TG × LDL-C/HDL-C; TG, triglyceride; LDL-C, low-density lipoprotein cholesterol; TMAO, trimethylamine N-oxide; TMA, trimethylamine; PLTP, phospholipid transfer protein; LCAT, lecithin–cholesterol acyltransferase; CETP, cholesteryl ester transfer protein; VLDL, very low density lipoprotein; AA, AA-genotype hens; AT, AT-genotype hens; TT, TT-genotype hens. Values are expressed as means ± SD, *n* = 6 hens each genotype. ^a,b^ Means within a row with no common superscript differ significantly (*p* < 0.05). ^A,B^ Means within a row with no common superscripts differ significantly (*p* < 0.01).

## Data Availability

The data that support the findings of this study are available on the request from the corresponding author. The data are not publicly available due to privacy or ethical restrictions.

## Supplementary Materials

The following supporting information can be downloaded at: https://www.mdpi.com/article/10.3390/ani12010048/s1, Table S1: The composition and nutrient level of basal diet.
