# Supplementary material for: T329S Mutation in the FMO3 Gene Alleviates Lipid Metabolic Diseases in Chickens in the Late Laying Period
Source: Animals (Basel). 2021 Dec 27;12(1):48. doi: 10.3390/ani12010048 (PMC8749748; doi:10.3390/ani12010048)
Supplement: Supplementary file 1 [file animals-12-00048-s001.zip › animals-1397625-supplementary.pdf]

Table S1. Composition and nutrient level of basal diet

Item	Basal Diet
Ingredient (%)	
Corn	61.95
Soybean meal	27.50
Soybean oil	0.75
Limestone	8.50
Sodium Salt	0.30
Cholesterol	-
Premix[1]	1.00
Total	100.00
Nutrient level[2] (%)	
ME (MJ/kg)	11.34
Crude protein	15.52
Lysine	0.71
Methionine	0.36
Threonine	0.66
Methionine + Cysteine	0.53
Calcium	3.40
Available phosphorus	0.28

[1]Premix provided the following per kilogram of diets: Vitamin A, 10000 IU; Vitamin D_3, 3000 IU; Vitamin E, 30 IU; Vitamin K_3 1 mg; Vitamin B_1, 1 mg; Vitamin B_2, 6 mg; Vitamin B_6, 3 mg; Vitamin B_{12}, 0.01 mg; biotin, 0.1 mg; folic acid, 0.3 mg; calcium pantothenate, 10 mg; niacin, 45 mg; Cu (as copper sulfate), 8 mg; Fe (as ferrous sulfate), 80 mg; Mn (as manganese sulfate), 100 mg; Zn (as zinc sulfate), 60 mg; I (as potassium iodide), 1 mg; Se (as sodium selenite), 0.3 mg.

[2]Crude protein and calcium levels were analyzed. Each value was based on triplicate determinations, but all other nutrient levels were calculated.
